# Anti-trypanosomal activity of non-peptidic nitrile-based cysteine protease inhibitors

**DOI:** 10.1371/journal.pntd.0005343

**Published:** 2017-02-21

**Authors:** Antonio C. B. Burtoloso, Sérgio de Albuquerque, Mark Furber, Juliana C. Gomes, Cristiana Gonçalez, Peter W. Kenny, Andrei Leitão, Carlos A. Montanari, José Carlos Quilles, Jean F. R. Ribeiro, Josmar R. Rocha

**Affiliations:** 1 Instituto de Química de São Carlos, Universidade de São Paulo, São Carlos, São Paulo, Brasil; 2 Faculdade de Ciências Farmacêuticas de Ribeirão Preto, Universidade de São Paulo, Ribeirão Preto, São Paulo, Brazil; 3 AstraZeneca, Mölndal, Sweden; 4 Grupo de Estudos em Química Medicinal – NEQUIMED, Instituto de Química de São Carlos – Universidade de São Paulo, São Carlos, São Paulo, Brazil; Ohio State University, UNITED STATES

## Abstract

The cysteine protease cruzipain is considered to be a validated target for therapeutic intervention in the treatment of Chagas disease. Anti-trypanosomal activity against the CL Brener strain of *T*. *cruzi* was observed in the 0.1 μM to 1 μM range for three nitrile-based cysteine protease inhibitors based on two scaffolds known to be associated with cathepsin K inhibition. The two compounds showing the greatest potency against the trypanosome were characterized by EC_50_ values (0.12 μM and 0.25 μM) that were an order of magnitude lower than the corresponding K_i_ values measured against cruzain, a recombinant form of cruzipain, in an enzyme inhibition assay. This implies that the anti-trypanosomal activity of these two compounds may not be explained only by the inhibition of the cruzain enzyme, thereby triggering a putative polypharmacological profile towards cysteine proteases.

## Author summary

Chagas disease is still considered a neglected tropical disease (NTD). There are more than 5 million people infected worldwide of which 99% are located in the Americas. Chagas disease has huge social and economic consequences for countries with significant proportions of their populations living in poverty. Chagas disease causes approximately seven thousand deaths per year and half a million people live with disabilities caused by the disease. Based on disability-adjusted life-years (DALYs), the disease burden of Chagas disease is five times greater than malaria and is approximately one-fifth of HIV/AIDS in the Latin American and Caribbean region. Despite being characterized over a century ago by Carlos Chagas who identified *Trypanosoma cruzi* as the causative agent, treatment of the disease is restricted to just two drugs (benznidazole and nifurtimox) that are effective only in the acute phase of the disease. Failure to rapidly diagnose *T*. *cruzi* infections and a poor side effect profile that causes many patients to abandon treatment both limit the effectiveness of treatment in the acute phase Chagas disease and many patients ultimately progress to the chronic phase. In this study, we have identified three compounds with anti-trypanosomal effects on the infective *T*. *cruzi* CL Brener form prevalent in various regions of the Americas, with potency in the 0.1 μM to 1 μM range and minimal cytotoxicity, even at 128 μM. Additionally, two of these compounds are significantly more potent against the parasite than against the recombinant form of the cysteine protease cruzipain which is generally considered to be a valid target for therapeutic intervention in the treatment of Chagas disease. These observations raise questions about the relevance of cruzain inhibition as a predictor of anti-trypanosomal activity and reinforce the case for using phenotypic assays in the search for new antichagasic agents. Correlation between enzyme inhibition and activity in cell-based assays is a general issue in drug discovery and we discuss the importance of intracellular unbound concentration in this context. We believe that this study is of significant interest both on account of the potent anti-trypanosomal activity observed for three of the compounds studied and the weak link between this activity and cruzain inhibition.

## Introduction

**Fig 1:**
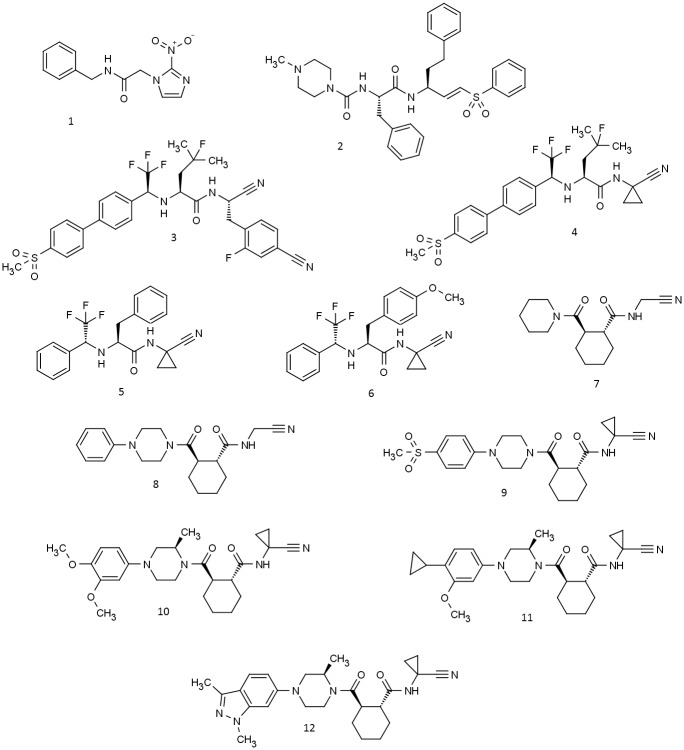
Known antichagasic agents (1–3) and cysteine protease inhibitors evaluated in the current study (4–12).

Chagas disease, also known as American trypanosomiasis, is a significant public health problem in Latin America [1–3]. Although considered to be a neglected tropical disease (NTD), Chagas disease is becoming more prevalent outside Latin America due to increased migration [4]. Chagas disease is caused by the protozoan parasite *T*. *cruzi*, which is transmitted by blood-sucking reduviid bugs of the subfamily Triatominae [1–3]. Current therapy is limited to benznidazole (**1**; chemical structures for compounds discussed in this study are presented in Fig 1) or nifurtimox, both of which are associated with side effect profiles that may impair the therapy and are only effective in the acute phase of the disease [2,5,6]. Recently, the antifungal drug posaconazole showed limited curative potential in a randomized clinical trial against chronic *T*. *cruzi* infection [7] and this has stimulated debate [8,9] about the extent to which the result might have been predicted using *in vivo* imaging. The paucity of therapeutic options for chronic Chagas disease has fueled interest in the discovery of new macromolecular targets and led to collaborative efforts worldwide, including initiatives such as Drugs for Neglected Diseases (DNDi) [10].

Cruzipain, also known as GP57/51, is the major cysteine protease of *T*. *cruzi* and is considered to be a validated target for therapeutic intervention in the treatment of Chagas disease [11–16]. The oral activity observed [13,15] for the cysteine protease inhibitor K777 (**2**) in animal disease models provides some of the basis for target validation. The natural enzyme is expressed as a mixture of isoforms, which differ in substrate preference and susceptibility to inhibitors, and consists of a catalytic domain linked to a carboxy-terminal extension which is retained in the mature protein [14,17]. Cruzipain is differentially expressed in the main stages of the parasite’s life cycle and is subject to extensive post-translational modification, mainly at sites in the carboxy-terminal extension [14]. Asn 33 in the catalytic domain of the mature enzyme is reported to be always glycosylated *in vivo* but this glycosylation site is absent in the cruzipain 2 isoform [14].

The activity [18] in a murine Chagas disease model of **3**, a structurally-elaborated analog of the cathepsin K inhibitor odanacatib (**4**) [19], the clinical development of which was recently discontinued in Phase III, provided the motivation for the current study. One objective was to gain a greater understanding of structure-activity relationships (SAR) for compounds based on the odanacatib scaffold. To this end, cruzain inhibition and anti-trypanosomal activity were characterized for odanacatib (**4**) and two of its analogues that were synthesized as part of this study to build on the extensive structure activity relationships reported for this scaffold [20]. Another objective was to evaluate cathepsin K inhibitors based on a cyclohexane dicarboxamide scaffold with respect to both cruzain inhibition and anti-trypanosomal activity.

During the course of this study, it was observed that the anti-trypanosomal activity of two of the compounds was significantly greater than what would be expected on the basis of their cruzain inhibitory activity. This leads us to pinpoint that reduction in potency when going from enzyme to cell inhibition is not observed for these compounds, and demonstrates that it is not the result of non-specific cytotoxicity.

## Materials and methods

### Ethics statement

The Ethics Committee on Animal Experimentation of the Faculty of Pharmacy of Ribeirao Preto–University of Sao Paulo, approved the cytotoxicity assays (approval no. 010263/2014). This Committee adheres to Conselho Nacional de Controle de Experimentação Animal–CONCEA, created by Brazilian Law number 11794 of 8 October 2008. Assays were run according to the guidelines of the Ministry of Science, Technology and Innovation of Brazil. The Biosafety Committee of the Faculty of Pharmacy of Ribeirao Preto–University of Sao Paulo, also approved the use of genetic modified organisms (approval no. 0019–17).

### Enzyme kinetic assays

Recombinant cruzain, consisting of the catalytic domain of cruzipain but excluding the carboxy-terminal extension, was expressed and purified as previously described [21]. Enzyme kinetic assays for cruzain were carried out at 37°C in 200 μL of a solution containing 100 mM acetate buffer pH 5.5, 300 mM NaCl, 5 mM dithiothreitol (DTT), 5% v/v dimethyl sulfoxide (DMSO), 0.01% v/v Triton X-100 and 0.15 nM cruzain, using Corning^®^ 96-well black flat bottom microplates. (S)-N-(1-((cyanomethyl)amino)-1-oxo-3-phenylpropan-2-yl)benzamide was used as a positive control. The rate of the reaction was monitored using a Biotek Synergy HT plate reader through the fluorescence emission at 460 nm (excitation at 355 nm) due to the hydrolysis of the substrate Z-Phe-Arg-7-amido-4-methylcoumarin (Z-FR-AMC, Sigma-Aldrich). The enzyme stock aliquot was rapidly thawed at 37°C and kept on ice until activation, in which it was incubated for 20 min. in the assay buffer (100 mM acetate pH 5.5 and 5 mM DTT) followed by additional 2 min. incubation with inhibitors before the reaction was started by the addition of the substrate.

Visual inspection and a pre-reading of plate wells were performed to check for possible precipitation and background fluorescence, respectively. Fluorescence emission spectra were also recorded for all inhibitors, using the same excitation wavelength (355 nm) as for the fluorometric assay. None of the compounds displayed a significant fluorescence signal around 460 nm, the emission wavelength used to monitor the reaction kinetic. Thus, potential inner-filter effects did not have to be taken into account in our experiments. The reaction was started by the addition of varying concentrations of the substrate and the wells monitored for a total of 5 minutes of reaction. The initial velocities of the substrate hydrolysis under first-order reaction were calculated by Gen5 Biotek software based on the linear-regression coefficient from the data fitting of Relative Fluorescence Unit (mRFU) as a function of time (min).

Each experiment was performed in duplicates for each compound being tested. A control measurement in absence of inhibitor (K_M_) was carried out for each setup plate and the kinetic affinity constants (K_i_) were calculated from non-linear fit of Michaelis-Menten curves to the data of initial velocities as a function of eight different concentrations of the substrate Z-FR-AMC from 30.0–0.8 μM. All inhibitors were evaluated in two different concentrations, which were chosen based on a previous screening (percentage of inhibition) at 1.7 μM substrate (~K_M_). SigmaPlot (v. 10.0) was employed for the non-linear fit and the kinetic parameters determination.

Enzyme kinetic assays for cathepsin L were performed in a similar manner to the cruzain assays at 37°C in 200 μL of a solution containing 100 mM acetate buffer pH 5.5, 300 mM NaCl, 1 mM dithiothreitol (DTT), 1 mM EDTA, 5% v/v dimethyl sulfoxide (DMSO), 0.01% v/v Triton X-100 and 0.30 nM cathepsin L, using Corning^®^ 96-well black flat bottom microplates. Using the QFRET technology, compounds 9–12 were tested for inhibition of cathepsin L-mediated cleavage of a synthetic peptide Z-Phe-Arg-AMC (10 μM final concentration) using 0.1 nM [final] human Cat L (prepared in-house) [24]. A potassium phosphate buffer was used to maintain pH at 6.4.

### Assays for anti-trypanosomal activity and cytotoxicity

Assays were performed against amastigote forms of *T*. *cruzi* CL Brener strain, which expresses β-galactosidase derived from *Escherichia coli* [22], infecting monkey kidney (LLC-MK2) cells as described [21]. Previously, LLC-MK2 host cells were cultivated in 96-wells microplates (10^3^ cells/well). After 12 hours, the trypomastigote form of CL Brener (B5 clone) strain was added in a 1:10 ratio and incubated for another 2 hours. Then, the wells were washed with PBS to remove the extracellular parasites and the tested compounds were added in the final concentrations of 0.031, 0.125, 0.5, 2.0, 8.0, 32.0, 128 and 512 μM. After this, the microplates were incubated at 37°C, in CO_2_ atmosphere (5%), for 5 days. Then, 10 μL of FluoReporter lacZ/Galactosidase Quantitation Kit (Molecular Probes, ThermoFisher Scientific) were added and after 30 minutes the fluorometric reaction was read in a microplate reader (Synergy H1, Biotek) at 386 nm excitation and 448 nm emission. The metabolism of the substrate is directly correlated with the amount of β-galactosidase activity and, therefore, the number amastigotes. The percentage of activity for each compound was determined from the following formula: %activity = 100 –{[(X-PC)/(NC-PC)]x100}, where the optical density values of the samples (X), the positive controls (PC) and negative controls (NC) were used. Culture medium alone was used as positive control (PC) and cells with medium and 0.6% DMSO (v/v) as negative control (NC). All assays were performed in triplicate with two independent experiments.

All compounds were subjected to the MTT colorimetric assay using Balb/C 3T3 clone A31 cells (mouse fibroblast cells) acquired from the Rio de Janeiro Cell Bank (BCRJ code 0047). Inhibition values were determined in the cell-based assay as previously described [23]. Briefly, cells were cultured at 37°C in an atmosphere of 5% CO_2_ using DMEM medium (Cultilab, Campinas-SP, Brazil) supplemented with 3.5 g glucose (Sigma-Aldrich), 1% penicillin/streptomycin solution and 10% FBS (Cultilab). Cells were plated at a concentration of 10^5^ cell/mL in 96-well plates and incubated for 24 h. All compounds were freshly diluted from 50 mM DMSO stock solutions to obtain the final concentration of 250 μM and added to each well by replacing the medium. The cell viability was assessed during 24, 48 and 72 h using the MTT (Sigma-Aldrich) reagent, with an incubation time of 3 h. Formazan crystals were dissolved using a solubility reagent composed of DMSO, glacial acetic acid and extran for 1 h. The readout was obtained using Biotek Synergy HT plate reader at 570 nm. Benznidazole was used as control. This assay was done in quadruplicate in two independent experiments. Statistical analyses were made in GraphPad Prism 5. Concentration-response curves were fitted for the best compounds and the statistical parameters are provided with the graphs in S9 Fig in the supplemental material.

### Synthetic chemistry

#### General synthetic procedures

Commercially available reactants and solvents were used as received, without further purification, unless otherwise stated. All solvents were dried and distilled prior to use by standard procedures. Reaction progress was monitored by thin layer chromatography on silica gel (aluminum foils) and spotted under UV light (254 nm), followed by staining with ethanolic 25% phosphomolybdic acid solution or with aqueous potassium permanganate.

Purification by column chromatography was carried out on silica gel (Merck 60, particle size 0.040–0.063 mm). Melting points were determined in a Kofler apparatus. Infrared spectra were obtained using FTIR at 4.0 cm^-1^ resolution and are reported in wavenumbers. ^1^H-NMR spectra were recorded at 400 MHz and the ^13^C-NMR spectra at 100 MHz, in CDCl_3_ or *d*^6^-DMSO at room temperature, in a Varian Mercury 400. Chemical shifts (δ) were reported in ppm (relative to TMS) and the coupling constants (J) in Hertz (Hz). ^1^H-NMR spectra were referenced to DMSO (δ = 2.54 ppm) or CDCl_3_ (δ = 7.23 ppm) as internal standard, and ^13^C-NMR were referenced to the central signal of the *d*^6^-DMSO multiplet (δ = 40.45 ppm) or CDCl_3_ triplet (δ = 77.00 ppm). Signal multiplicity was assigned as singlet (s), doublet (d), double doublet (dd), triplet (t), double triplet (dt), quartet (q), quintuplet (qt), multiplet (m) and broad (br). The high resolution mass spectra (HRMS) were recorded using a Q-TOF Micromass equipment (Waters, UK) by means of ESI-MS techniques.

#### Synthesis of compound 5

*(S)-methyl 2-amino-3-phenylpropanoate*. L-Phenylalanine (18.2 mmol, 3.0 g, 1 equiv,) was suspended in methyl alcohol (21 ml), and cooled to 0°C. Thionyl chloride (20.0 mmol, 1.45 ml, 1.1 equiv.) was added slowly, over a period of 20 min and the reaction mixture was then heated on a steam bath for 1h and afterward at ambient temperature overnight. Dry ether was added to the solution until turbidity appeared. The mixture was then refrigerated for several hours, during which time the methyl ester crystallized. The product was collected by filtration to afford the aminoester in 88% yield. White solid, Mp 156–160°C. FT-IR (KBr, *ʋ*_max_): 3439, 2847, 2623, 1747, 1583, 1497, 1242, 1146, 742 cm^-1^. ^1^H NMR (400 MHz, CDCl_3_): *δ* = 3.10 (dd, 7.69 and 13.93Hz, 1H), 3.24 (dd, *J* = 5.51 and 13.94Hz, 1H), 3.63 (s, 3H), 4.19 (dd, *J* = 5.59 and 7.62Hz, 1H), 7.23–7.36 (m, 5H) 8.86 (s, 2H) ppm. ^13^C NMR (100 MHz, CDCl_3_): δ = 36.2, 52.9, 53.7, 127.7, 129.0, 129.8, 135.2, 169.7 ppm. HRMS (ESI): m/z calcd for C_10_H_14_NO_2_ [M + H]^+^: 180.10191; found: 180.10178.

*(2S)-3-phenyl-2-((2*,*2*,*2-trifluoro-1-phenylethyl)amino)propanoic acid*. To an (*S*)-methyl 2-amino-3-phenylpropanoate solution in dry methanol (0.47 M), under argon atmosphere, potassium carbonate (5.2 mmol, 727 mg, 0.94 equiv.) and freshly prepared (ref 2) 2,2,2-Trifluoroacetophenone (6.1 mmol, 0.86 mL, 1.1 equiv.) were added. The mixture was stirred at 50°C for 18 h and then the reaction was filtered and the solvent was removed in vacuum. The collected oil was subsequently washed with tert-butyl methyl ether (3 x 10 mL) and dried under vacuum until constant mass. The products isolated in this way were essentially pure to be used in the next step without chromatographic purification. The potassium (S,Z)-3-phenyl-2-((2,2,2-trifluoro-1-phenylethylidene) amino)propanoate (1.4 mmol, 500 mg, 1 equiv.) was dissolved in dry THF (10mL), stirred at 0°C and then, NaBH_4_ (5.6mmol, 210 mg, 4 equiv.) was added in one batch. After stirring for 4h, at room temperature, the system was cooled to 0°C and then a 2 M HCl solution was added dropwise until stopping the gas release. Next, the solvent was evaporated under vacuum and the crude extracted with ethyl acetate (3 x 10 mL). The combined organic layers were then dried over Na_2_SO_4_, filtered, and evaporated in rotary evaporator. Purification by flash column chromatography (30% AcOEt/Hexane) afforded (2S)-3-phenyl-2-((2,2,2-trifluoro-1-phenylethyl)amino)propanoic acid in 67% yield. White solid. Mp 160–163°C. FT-IR (KBr, *ʋ*_max_): 3306, 3242, 2927, 2530, 1720, 1470, 1261, 1221, 894, 619 cm^-1^. ^1^H NMR (400 MHz, CDCl_3_): *δ* = 2.81(dd, *J* = 8.10 and 13.43 Hz, 1H), 2.89 (dd, *J* = 5.40 and 13.44 Hz, 1H), 3.06–3.09 (m, 1H), 4.41 (q, *J* = 7.67 Hz, 1H), 7.12–7.41 (m, 10H) ppm. ^13^C NMR (100 MHz, CDCl_3_): δ = 39.0, 59.6, 62.1 (q, *J* = 27.94 Hz, 1C), 122.4, 124.6, 126.8, 126.9, 128.4, 128.8, 129.3, 129.4, 129.8, 133.7, 138.2, 138.3, 174.7, 174.8 ppm. HRMS (ESI): m/z calcd for C_17_H_17_F_3_NO_2_ [M + H]^+^: 324.12059; found: 324.12067.

*(S)-N-(1-cyanocyclopropyl)-3-phenyl-2-(((R)-2*,*2*,*2-trifluoro-1-phenyl-ethyl)amino)propanamide (5)* (2S)-3-phenyl-2-((2,2,2-trifluoro-1-phenylethyl)amino)propanoic acid (0.31 mmol, 100 mg, 1 equiv.), 1- [Bis(dimethylamino)methylene]-1H-1,2,3-triazol[4,5-b]pyridinium-3-oxido hexafluorophosphate (HATU, 0.46 mmol, 176 mg, 1.5 equiv) and 2-cyclopropylaminonitrile (0.34 mmol, 40 mg, 1.1 equiv.) were sequentially introduced into a 10 mL round-bottomed reaction flask, provided with magnetic stirring and under argon atmosphere. Next, dry DMF (1mL) and N,N-Diisopropylethylamine (0.46 mmol, 80μL, 1.5 equiv.) were added into the flask. The reaction was stirred at room temperature overnight. The disappearance of the starting trifluoromethyl amino acid was confirmed by TLC monitoring. When the reaction was complete, ethyl acetate (10 ml) was added to the reaction and the organic layer was washed with saturated aqueous NaCl solution (10x10 ml). The organic layer was then dried over Na_2_SO_4_, filtered, and evaporated in rotary evaporator. Purification by flash column chromatography (30% AcOEt/Hexane) afforded the mixture of **5** and its diastereomer in 90% yield (*trans*:*cis* ratio = 95:5). The diasteromers were separated by HPLC (Phenomenex celulose II, 25cm x 4.6μm, Acetonitrile/Water: 60:40). 92% Yield. Colorless oil. [α]_D_^24^ = -59.57° (MeOH, c = 1,17x 10^−3^). FT-IR (KBr, *ʋ*_max_): 3329, 3246, 3032, 2237, 1649, 1539, 1499, 1456, 1263, 1171, 1126, 850, 742, 567 cm^-1^. ^1^H NMR (400 MHz, CDCl_3_): *δ* = 1.16–1.22 (m, 1H), 1.26–1.32 (m, 1H), 1.55–1.65 (m, 2H), 2.13 (s, 1H), 2.68 (dd, *J* = 9.87 and 13.86Hz, 1H), 3.14 (dd, *J* = 4.25 and 13.87Hz, 1H), 3.24 (dd, *J* = 4.25 and 9.85Hz, 1H), 3.74 (q, *J* = 7.73Hz, 1H), 6.81 (d, *J* = 7.52Hz, 2H), 7.19–7.33 (m, 6H), 7.71 (s, 1H) ppm. ^13^C NMR (100 MHz, CDCl_3_): δ = 16.4, 17.0, 20.1, 39.2, 63.6 (q, *J* = 63.59Hz), 119.7, 123.7, 125.9, 127.4, 127.8, 128.8, 129.0, 129.1, 129.2, 132.2, 135.7, 173.6 ppm. HRMS (ESI): m/z calcd for C_21_H_21_F_3_N_3_O [M + H]^+^: 388.16312; found: 388.16379. HPLC: Phenomenex celulose II, 25cm x 4.6μm, Acetonitrile/Water: 50:50, rt: 21.115 min, 206nm. Additional analytical data is provided as supplemental information: S1 and S2 Figs for ^1^H NMR, ^13^C NMR spectra; S5 and S6 Figs and S1 and S2 Tables for chromatograms of compound **5**.

#### Synthesis of compound 6

Compound **6** was prepared in an analogous manner to **5**

*S)-N-(1-cyanocyclopropyl)-3-phenyl-2-(((R)-2*,*2*,*2-trifluoro-1-(4-methoxy-phenyl)ethyl)amino)propanamide (6)*:

Colorless oil. [α]_D_^24^ = -5.68° (MeOH, c = 1,76x 10^−3^). FT-IR (KBr, *ʋ*_max_): 3273, 3038, 2237, 1672, 1610, 1533, 1512, 1265, 1145, 1039, 873, 794, 561 cm^-1^. ^1^H NMR (400 MHz, CDCl_3_): *δ* = 1.17–1.32 (m, 2H), 1.55–1.64 (m, 2H), 2.08 (s, 1H), 2.63 (dd, *J* = 9.75 and 14.03 Hz, 1H), 3.07 (dd, *J* = 4.27 and 14.02 Hz, 1H), 3.20 (dd, *J* = 4.27 and 9.73 Hz, 1H), 3.73 (q, *J* = 7.70Hz, 1H), 3.83 (s, 3H), 6.75–6.95 (m, 6H), 7.21–7.24 (m, 2H), 7.31–7.34 (m, 1H) ppm. ^13^C NMR (100 MHz, CDCl_3_): δ = 16.4, 17.0, 20.1, 38.3, 55.3, 63.66 (q, *J* = 29.20Hz), 114.4, 119.7, 123.7, 125.9, 127.5, 127.8, 128.8, 129.2, 130.0, 132.3, 158.9, 173.7 ppm. HRMS (ESI): m/z calcd for C_22_H_23_F_3_N_3_O_2_ [M + H]^+^: 418.17369; found: 418.17435. HPLC: Phenomenex celulose II, 25cm x 4.6μm, Acetonitrile/Water: 50:50, rt: 25.261 min, 206nm. Additional analytical data is provided as supplemental information: S3 and S4 Figs for ^1^H NMR, ^13^C NMR spectra; S7 and S8 Figs and S3 and S4 Tables for chromatograms of compound **6**.

## Results

**Fig 2:**
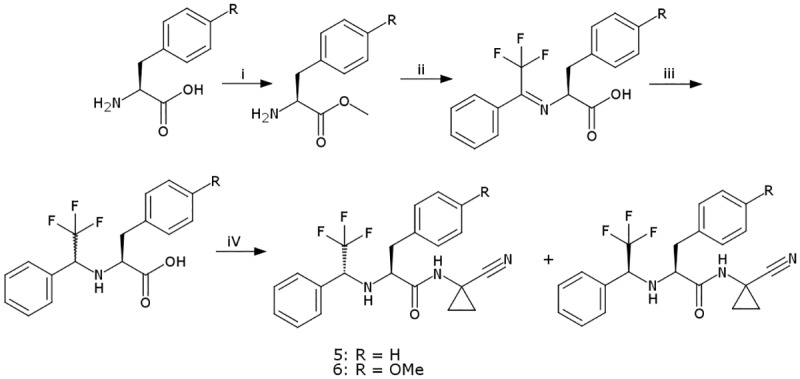
Synthesis of compounds 5 and 6. *Reagents and conditions*: (i) SOCl_2_, MeOH, r.t., 2.5 h, reflux 30 min, r.t. 4 h, 88%; (ii) PhC (= O)CF_3_, K_2_CO_3_, MeOH, 50°C, 18 h, R = H 66%, R = OCH_3_ 58%; (iii) THF, NaBH_4_, R = H 67%, R = OCH_3_ 56%; (iv) HATU, 1-amino-1-cyclopropanecarbonitrile, DIPEA, DMF, r.t., 24 h, R = H 92%, R = OCH_3_ 90%.

A number of the compounds in this study are known to be potent inhibitors of cathepsin K. Compound **4** (odanacatib [19]) is a cathepsin K inhibitor that was recently discontinued from advanced clinical development. Compound **3** was the result of optimization [18,20] of **4** with respect to cruzain inhibition although neither this nor the anti-trypanosomal activity of **4** appear to have been reported previously. Compounds **5** and **6**, in which the S3 substituent of **4** has been deconstructed, were synthesized (Fig 2) during the course of this study. Compounds **7**–**12** are published [24,25] cathepsin K inhibitors based on a cyclohexane dicarboxamide scaffold. This set of six probes was selected to include both structural prototypes (e.g. **7** and **8**) [23] and compounds (e.g. **10**, **11** and **12**) [25] for which pharmacokinetic data are available. Measured data including IC_50_ values against a number of cathepsins, aqueous solubility, logD_7.4_ and microsomal stability have been published for all six compounds [24,25].

Inhibition of cruzain and a number of cathepsins are reported in Table 1 (see Fig 1 for structures). We report enzyme inhibitory activity as pK_i_ or pIC_50_ which facilitates SAR perception and provides a more appropriate representation of measurement precision than do K_i_ or IC_50_. With the exception of odanacatib (**4**), none of the compounds show sub-micromolar potency against cruzain. Compounds **5** and **6** are slightly more potent against cathepsin L than cruzain and for the other compounds this trend is reversed. Compounds **4** and **7**–**12** are all two to three orders of magnitude more potent against cathepsin K than against cruzain.

**Table 1:** Inhibition of cruzain and cathepsins by nitrile-based cysteine protease inhibitors.

Compound	Cruzain pK_i_ ^a^^,^^b^	Cathepsin L pK_i_ ^a^^,^^b^	Cathepsin L pIC_50_ ^c^	Cathepsin K pIC_50_ ^c^	Cathepsin B pIC_50_ ^c^	Cathepsin S pIC_50_ ^c^
**4**	6.9 (0.09)	5.2 (0.07)	5.5 ^d^	9.7 ^d^	6.0 ^d^	7.2 ^d^
**5**	5.6 (0.07)	6.1 (0.04)				
**6**	5.1 (0.05)	5.8 (0.04)				
**7**	5.1 (0.02)		< 4 ^e^	7.1 ^e^	5.1 ^e^	5.7 ^e^
**8**	5.3 (0.02)		< 4 ^e^	8.1 ^e^	6.3 ^e^	6.0 ^e^
**9**	5.3 (0.09)		< 5 ^f^	7.7 ^g^	5.6 ^g^	5.7 ^g^
**10**	5.9 (0.06)		< 5 ^f^	8.4 ^g^	5.7 ^g^	5.8 ^g^
**11**	5.6 (0.06)		< 5 ^f^	8.5 ^g^	5.9 ^g^	5.9 ^g^
**12**	5.5 (0.07)		< 5 ^f^	8.0 ^g^	6.1 ^g^	5.9 ^g^

^a^ pK_i_ = −log_10_(K_i_/M)

^b^ Uncertainty shown in parentheses

^c^ pIC_50_ = −log_10_(IC_50_/M)

^d^ Reference [19]

^e^ Reference [24]

^f^ Determined using the Cat L inhibition assay method described in ref. [24]

^g^ Reference[25]

**Fig 3:**
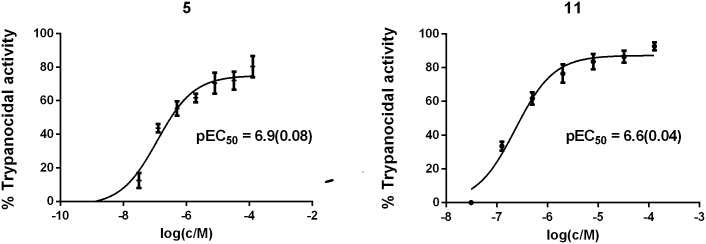
Concentration-response of anti-trypanosomal activity for compounds 5 and 11 (c = concentration of compound; M = mol/dm^3^).

Anti-trypanosomal activity (as pEC_50_ values) and cytotoxicity are presented in Table 2 for the compounds and data for benznidazole (**1**) are also included for comparison. It was not possible to derive meaningful EC_50_ values for the less potent compounds and % anti-trypanosomal activity in the concentration range 0.125 μM to 8 μM is also listed in order to allow comparisons to be made with the less potent compounds. Concentration-responses for anti-trypanosomal activity are shown in Fig 3 for the two most potent compounds (**5** and **11**). It was not possible to determine meaningful CC_50_ values for any of the compounds because only low levels of cytotoxicity were observed for LLC-MK2 and Balb/C 3T3 clone A31 cells at the highest concentration (128 μM) in the assays.

**Table 2:** Anti-trypanosomal activity of cysteine protease inhibitors.

Compound	%activity ^a^ at 8 μM	%activity ^a^ at 2 μM	%activity ^a^ at 0.5 μM	%activity ^a^ at 0.125 μM	pEC_50_ ^b^	Cytotox LLC-MK2 ^c^	Cytotox Balb/C 3TC ^d^
**1** ^e^	59 (3)	33 (3)	7 (3)	3 (2)	5.6 (0.04)	41 (8)	0 (0)
**4**	44 (2)	36 (5)	28 (2)	22 (4)	5.6 (0.08)	31 (4)	27 (15)
**5**	70 (6)	62 (3)	55 (4)	44 (2)	6.9 (0.08)	0 (0)	1 (7)
**6**	22 (2)	17 (4)	1 (2)	0 (0)	4.9 (0.08)	0 (0)	19 (6)
**7**	12 (4)	9 (1)	2 (1)	0(0)	N/A	4 (3)	2 (7)
**8**	35 (2)	21 (3)	7 (3)	1 (0.4)	N/A	29 (8)	0 (0)
**9**	33 (5)	30 (4)	15 (3)	6(2)	N/A	0 (0)	3 (7)
**10**	28 (5)	26 (6)	22 (3)	6(1)	N/A	15(3)	0 (0)
**11**	83 (5)	76 (5)	62 (4)	33 (3)	6.6 (0.04)	36 (7)	0 (0)
**12**	50 (4)	45 (3)	40 (5)	20 (2)	5.9 (0.06)	8 (2)	0 (0)

^a^ %activity; standard deviation (N = 6) in parentheses

^b^ pEC_50_ = −log_10_(EC_50_/M)

^c^ % Cytotoxicity in LLC-MK2 cells at 128 μM; standard deviation (N = 6) in parentheses

^d^ % Cytotoxicity in Balb/C 3T3 clone A31 cells at 128 μM; standard deviation (N = 8) in parentheses

^e^ Anti-trypanosomal activity of the antichagasic drug benznidazole is included for comparison purposes

## Discussion

**Fig 4:**
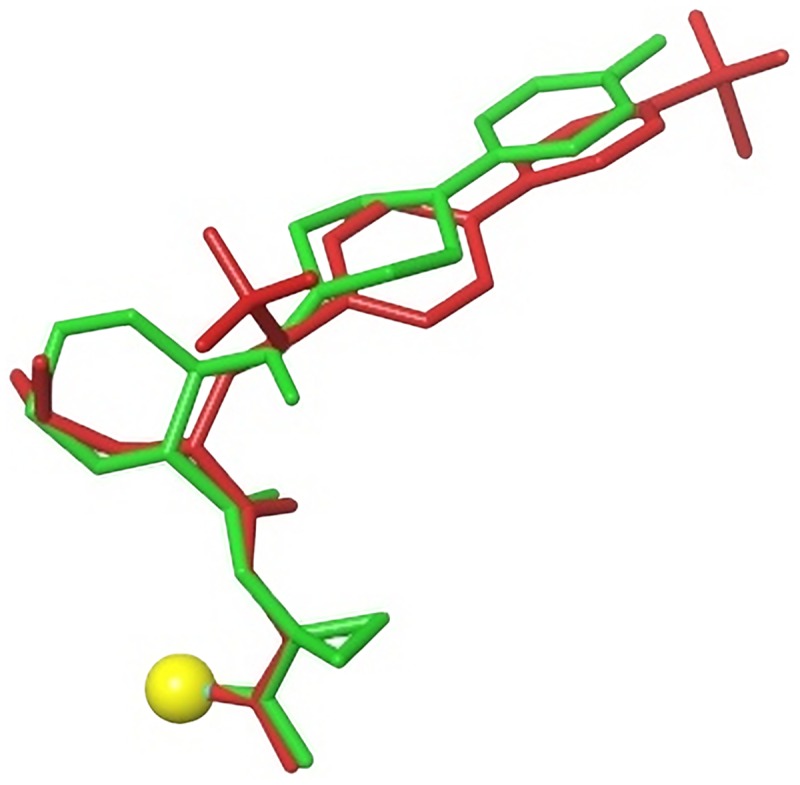
Overlay of cathepsin K inhibitors based on cyclohexane dicarboxamide (green) and odanacatib (red) scaffolds generated by alignment of Protein Data Bank (www.rcsb.org) [26] entries 1VSN [27] and 4DMX [24]. Cysteine sulfur with which the nitrile carbon forms a covalent bond is shown as a yellow sphere.

The published [18] efficacy of **3** and structural analogs in a murine Chagas disease model provided a motivation for the current study. Previously, we reported [21] that potent dipeptidyl nitrile inhibitors of cruzain did not show significant anti-trypanosomal activity and questioned the relevance of potency in this enzyme kinetic assay as a predictor for anti-trypanosomal activity. In searching for alternatives to the dipeptidyl scaffold, we observed that cathepsin K inhibitors based on cyclohexane dicarboxamide and odanacatib scaffolds bound to their target in a similar manner (Fig 4). These both differ from the dipeptidyl scaffold used in our previous study in that they lack the secondary amide linking the S3 substituent to the scaffold and are arguably less peptidic. The scaffold on which odanacatib is based allows easier access to the S2 region of the binding site than does the cyclohexane dicarboxamide scaffold.

Compound **4** (pK_i_ = 6.9; pEC_50_ = 5.6) was the most potent cruzain inhibitor of the compounds assayed and its pEC_50_ value is the same as that measured for benznidazole (1; pEC_50_ = 5.6). The anti-trypanosomal activity of 5 (pK_i_ = 5.6; pEC_50_ = 6.9), 11 (pK_i_ = 5.6; pEC_50_ = 6.6) and 12 (pK_i_ = 5.5; pEC_50_ = 5.9) exceeds the acceptability threshold recommended [28] for hits by one to two orders of magnitude even though these compounds are all less potent cruzain inhibitors than **4**. Compounds **5** (pEC_50_ − pK_i_ = 1.3), **11** (pEC_50_ − pK_i_ = 1.0) and **12** (pEC_50_ − pK_i_ = 0.4) all show higher levels of anti-trypanosomal activity than might be expected on the basis of their cruzain inhibition. While it was not possible to determine meaningful pEC_50_ values for every compound, structure-activity relationships (SAR) for anti-trypanosomal activity can still be discerned. For example, **5** is the most potent of the compounds based on the odanacatib scaffold despite having the lowest molecular weight. Potency tends to increase with molecular size for compounds in structural series so this observation is noteworthy. The contrasting profiles of **4** (pK_i_ = 6.9; pEC_50_ = 5.6) and 5 (pK_i_ = 5.6; pEC_50_ = 6.9) may also reflect stereochemical differences since the configuration of the S3 substituent of the former is *S* while that of the latter is *R*. In contrast, the lower molecular weight compounds based on the cyclohexane dicarboxamide scaffold show lower levels of anti-trypanosomal activity than **11** (pK_i_ = 5.6; pEC_50_ = 6.6) or **12** (pK_i_ = 5.5; pEC_50_ = 5.9).

Although failure of potency against the enzyme to translate to potency against cells would usually be seen as a problem to be solved rather than a phenomenon to be explained, it is important to first articulate why a compound might be less potent in an enzyme inhibition assay than in a cell-based assay. In drug discovery programs, failure of potent enzyme inhibition to translate to cell-based potency is typically attributed to poor permeability. Slow diffusion of a drug into cells means that the intracellular unbound concentration of drug will be lower than its unbound concentration in assay buffer. It can be helpful to think of drug action as being driven by its Target Engagement Potential [29], one measure of which can be derived by scaling unbound concentration (C_u_) [30–32] by the dissociation constant (K_D_) for the complex between drug and target:

$$\mu_{\text{drug}}={\text{ log}}_{10}\left({\text{C}}_{\text{u}}/{\text{K}}_{\text{D}}\right)$$

In this framework, poor permeability can be thought of as preventing μ_drug_ from attaining levels required for cellular activity. Even when passively permeable, compounds may be substrates for transporters and subject to efflux [31,33,34,35]. However, a compound may also fail to show activity in a cell-based assay because the enzyme has a relatively high affinity for its substrate(s) and/or cofactor or that the latter species may be present at higher concentrations than in the enzyme kinetic assay. In these situations, a higher value of μ_drug_ is required for target engagement in the cell than in the enzyme kinetic assay and this is particularly relevant for kinase targets which are all exposed to the same intracellular concentration of adenosine triphosphate. Although intracellular unbound concentration of compounds can be measured [30–32] *in vitro* in some situations, *in vivo* measurement is not currently feasible and phase I clinical trials are typically incomplete when targets are intracellular [36]. This is a factor that needs to be considered when interpreting poor efficacy in a phase II trial as evidence that the target is not relevant to human disease. When the infectious agent itself is intracellular, as is the case for Chagas disease, leishmaniasis, malaria and tuberculosis, the uncertainty in physiological concentration is correspondingly greater and permeability requirements are likely to be even more stringent. In one scenario, transporter-driven efflux might lead to significant differences between drug concentration in the parasite and in the host cell (where off-target effects may lead to toxicity). Perturbation of host cell transporter expression by intracellular infectious agents should be anticipated as a parasite defense mechanism.

An increase in potency going from enzyme to cell assay, such as was observed for **5** and **11** is less commonly encountered in drug discovery projects. The primary inference that may be drawn from the observation that **5** and **11** are both less potent against the enzyme than against the parasite is that anti-trypanosomal activity of these compounds cannot be explained by their cruzain inhibition. The possibility of non-specific cytotoxicity is always a concern when compounds are more potent in a cell-based assay than in the corresponding enzyme inhibition assay. However, only low levels of cytotoxicity in two cell lines were observed for the compounds in this study at 128 μM (top concentration used in the cytotoxicity assay), which is almost three orders of magnitude greater than EC_50_ values for the two most potent compounds. It should be stressed that the primary purpose of the cytoxicity assay in a study like this is to show that the activity of compounds is not due to non-specific cytoxicity rather than to provide evidence that compounds are safe for dosing *in vivo*. It is also relevant that anti-trypanosomal activity is observed for compounds based on two distinct scaffolds and that the SAR is not ‘flat’. As noted previously, uncertainty in unbound concentration [30–32] at the site of action must always be considered when interpreting results of cell-based assay results, especially when the site of action is within an intracellular parasite. However, neither **5** nor **11** has a basic center that could cause it to concentrate [37] within acidic compartments in the trypanosomes and one would need to invoke transporter-mediated influx [31] in order to explain elevated free concentrations at the site(s) of action. It must be stressed that neither poor permeability nor substrate concentration/affinity can be invoked as an explanation for compounds showing greater potency in a cell-based assay than when assayed against the isolated enzyme.

The observation that **5** and **11** are an order of magnitude more potent in the assay for anti-trypanosomal activity than in the cruzain inhibition assay points to other plausible scenarios that are relevant to the search for effective antichagasic agents. The original validation of cruzipain as a target (and cruzain as a model for cruzipain) was performed using irreversible cysteine protease inhibitors such as peptidyl diazomethanes [11], peptide-fluoromethyl ketones [12] and vinyl sulfones such as **2** [13] that do not inhibit cruzain selectively. Compound **2** even shows efficacy in a murine model of schistosomiasis [38]. One interpretation of the results of the current study is that the anti-trypanosomal effects of the most potent compounds are due to inhibition of one or more cysteine proteases (including isoforms, such as cruzipain-2 for instance) that have not yet been identified as potential targets for therapeutic intervention. There is a possibility that compounds 5 and 11 may show a polypharmacological profile, targeting isoforms of cruzipain or other cysteine proteases present in the parasite. For example, the study of cruzipain-2 [39] highlights the necessity of a deeper understanding of the implications of a putative family-driven inhibitor instead of a highly selectivity one in terms of *T*. *cruzi* activity.

The cysteine protease inhibition profile (Table 1) suggests that a putative new antichagasic target may well share molecular recognition characteristics with cathepsin K and that phenotypic screening libraries for discovery of antichagasic agents should specifically include potent inhibitors of this enzyme.

Another possibility is that the unglycosylated catalytic domain of a single isoform used in the enzyme kinetic assay and for virtual screening [40,41] may, in some situations, be an inappropriate model for the trypanosomal enzyme, which is expressed as a mixture of isoforms and is subject to extensive post-translational modification. While ‘false positives’ are recognized [40] as a problem when screening against cruzain, a physiologically inappropriate target model can also lead to ‘false negatives’ which, by their nature, are less easily recognized. Even when a target has been validated, there may still be advantages in using a phenotypic [42,43] assay for discovery of compounds that show activity against the target and to assess SAR.

## Conclusions

We have demonstrated that compounds **5** (pEC_50_ = 6.9; EC_50_ = 0.12 μM), **11** (pEC_50_ = 6.6; EC_50_ = 0.25 μM) and **12** (pEC_50_ = 5.9; EC_50_ = 1.3 μM) are potent anti-trypanosomal agents. Given the current dearth of therapeutic options for this NTD, the anti-trypanosomal activity of these compounds is of considerable interest in its own right. However, the results also have broader relevance for antichagasic drug design because compounds **5** and **11** are an order of magnitude more potent against *T*. *cruzi* than against the recombinant form of cruzipain, which is generally considered to be a valid target. The results highlight two potential scenarios relevant to the discovery of new antichagasic agents. First, the anti-trypanosomal effects of the compounds could be due to engagement of one or more targets other than cruzipain. Secondly, the molecular recognition characteristics of cruzipain may either differ from cruzain or be altered by post-translational modification, which would highlight the need for new structural models for cruzipain. Knowledge of the activity of **5**, **11** and **12** could also be used to develop tools for the exploration of each scenario. The results of this study reinforce the case for using phenotypic assays in the search for new antichagasic agents even when the target is ‘known’.

## Supporting information

S1 Fig ^1^H NMR spectrum (400 MHz, CDCl_3_) for 5. (DOCX)

S2 Fig ^13^C NMR spectrum (100 MHz, CDCl_3_) for 5. (DOCX)

S3 Fig ^1^H NMR spectrum (400 MHz, CDCl_3_) for 6. (DOCX)

S4 Fig ^13^C NMR spectrum (100 MHz, CDCl_3_) for 6. (DOCX)

S5 Fig Chromatogram of mixture of 5 and its diasteromer. (DOCX)

S6 Fig Chromatogram after HPLC separation to compound 5. (DOCX)

S7 Fig Chromatogram of mixture 6 and its diastereomers. (DOCX)

S8 Fig Chromatogram after HPLC separation to compound 6. (DOCX)

S9 Fig Concentration response for trypanocidal activity. (DOCX)

S1 Table Chromatogram data for S5 Fig. (DOCX)

S2 Table Chromatogram data for S6 Fig. (DOCX)

S3 Table Chromatogram data for S7 Fig. (DOCX)

S4 Table Chromatogram data for S8 Fig. (DOCX)
