# Supplementary material for: Anti-trypanosomal activity of non-peptidic nitrile-based cysteine protease inhibitors
Source: PLoS Negl Trop Dis. 2017 Feb 21;11(2):e0005343. doi: 10.1371/journal.pntd.0005343 (PMC5344518; doi:10.1371/journal.pntd.0005343)
Supplement: S2 Fig — (DOCX) [file pntd.0005343.s002.docx]

**S2 Fig.** ^13^C NMR spectrum (100 MHz, CDCl_3_) for **5**
