# Supplementary figures and images for: Anti-trypanosomal activity of non-peptidic nitrile-based cysteine protease inhibitors
Source: PLoS Negl Trop Dis. 2017 Feb 21;11(2):e0005343. doi: 10.1371/journal.pntd.0005343 (PMC5344518; doi:10.1371/journal.pntd.0005343)

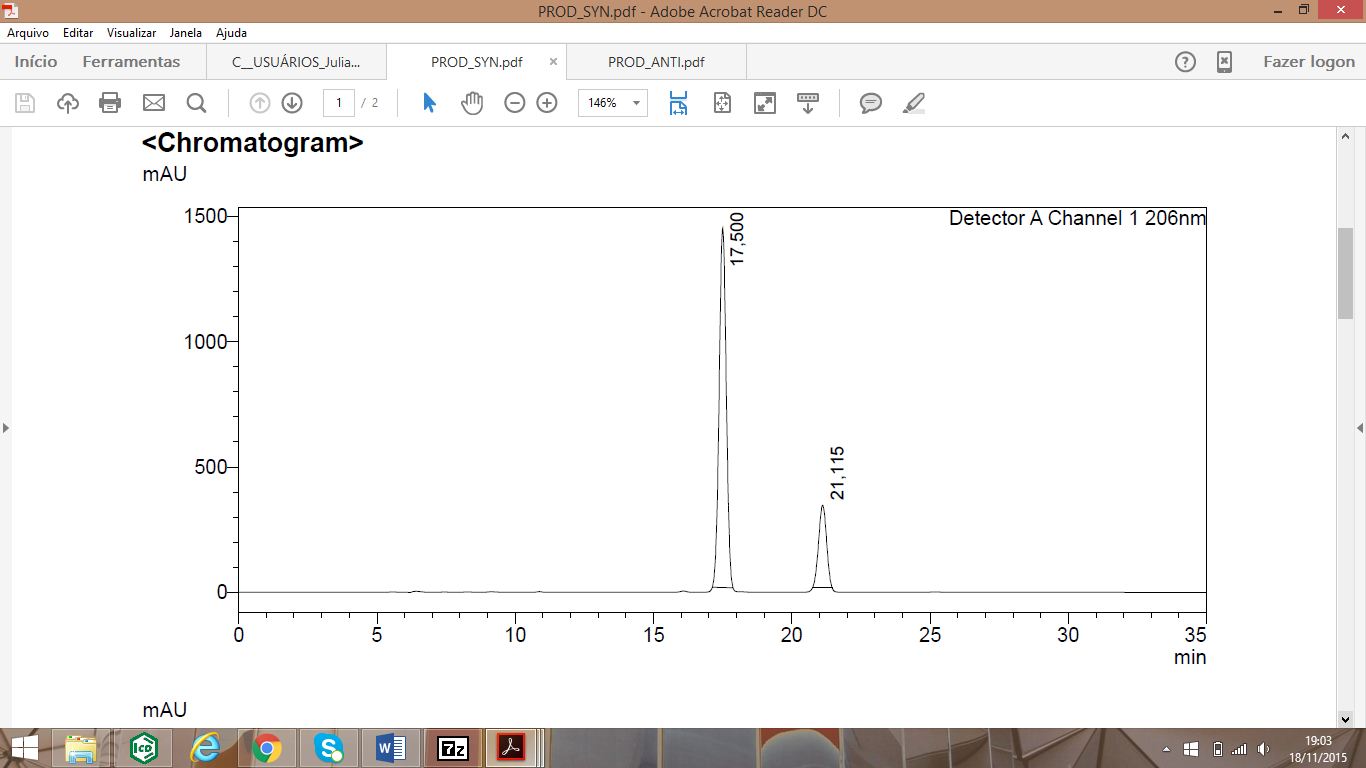


**S5 Fig.** Chromatogram of mixture of **5** and its diasteromer

Supplement: S5 Fig — (DOCX) [file pntd.0005343.s005.docx]

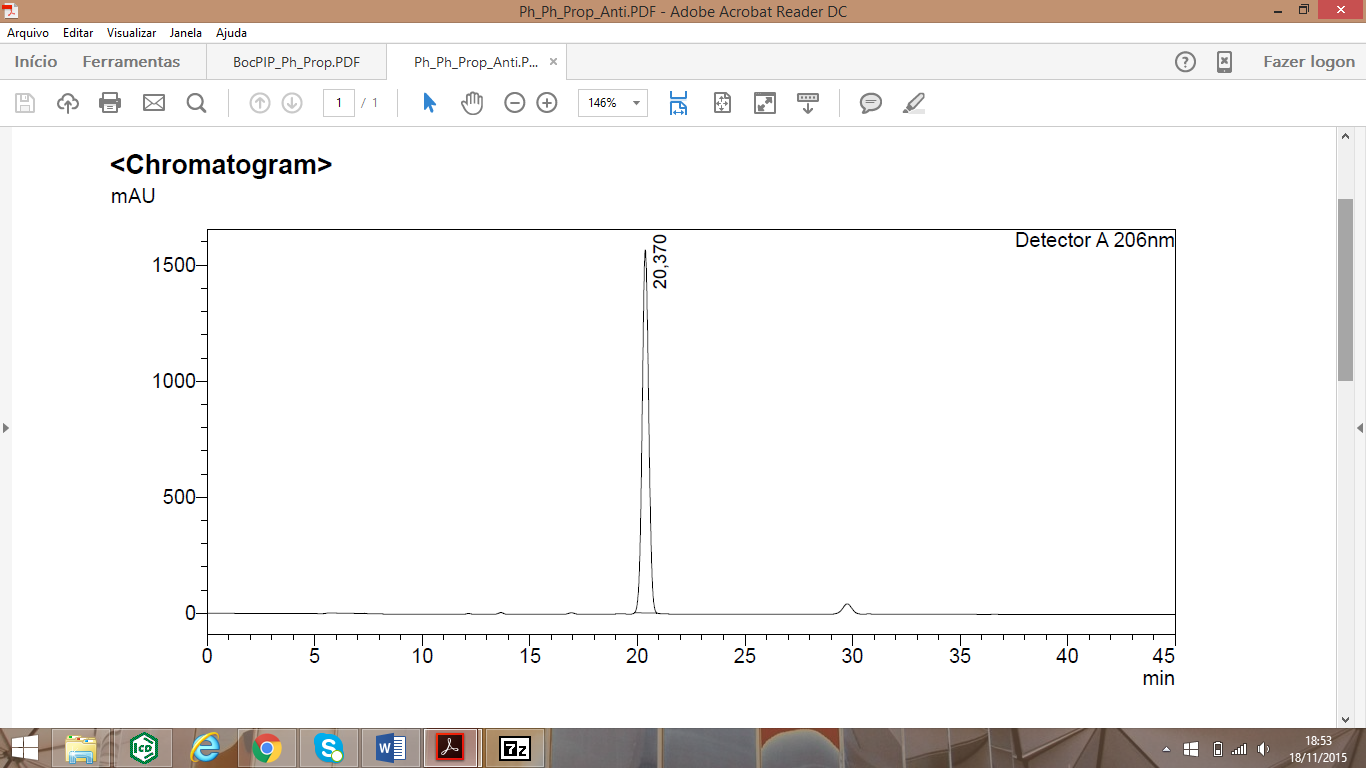


**S6 Fig.** Chromatogram after the HPLC separation to compound **5**

Supplement: S6 Fig — (DOCX) [file pntd.0005343.s006.docx]

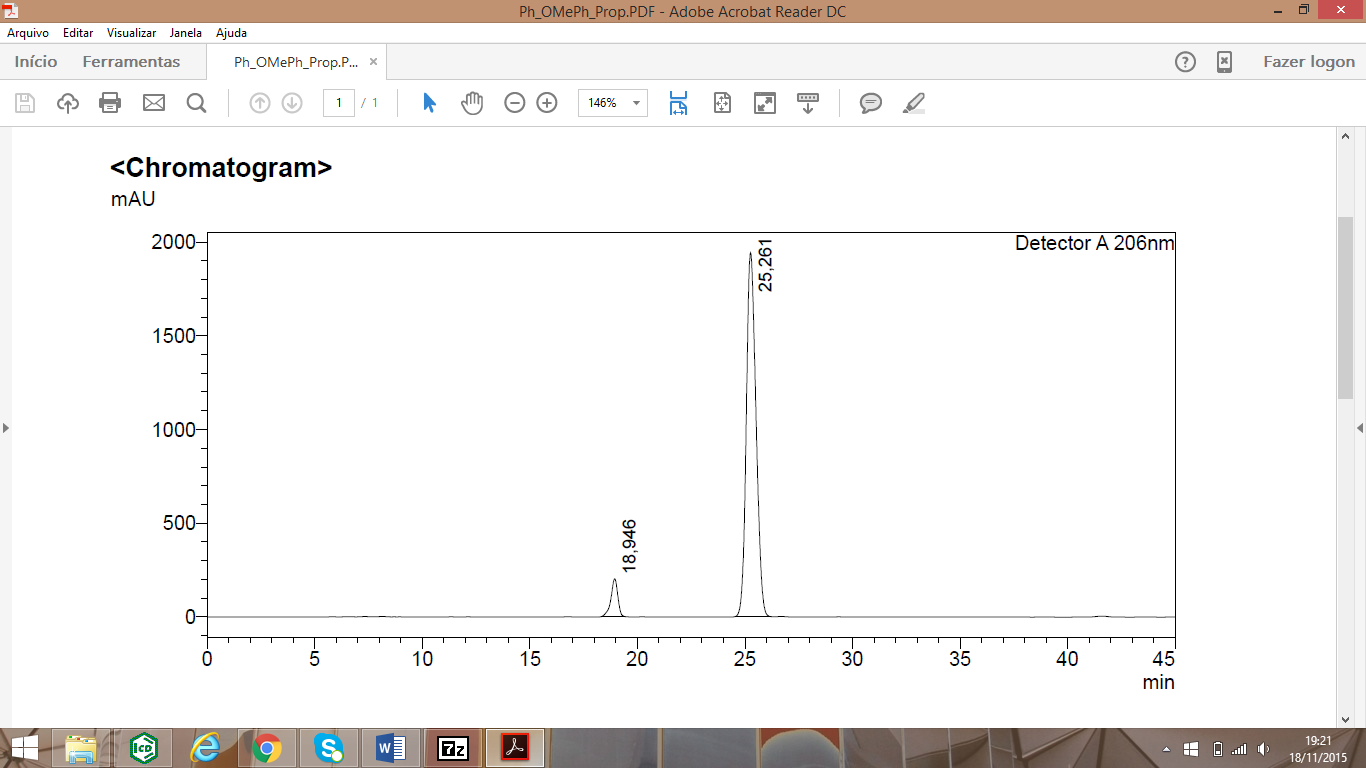


**S7 Fig.** Chromatogram of mixture **6** with its diastereomer

Supplement: S7 Fig — (DOCX) [file pntd.0005343.s007.docx]

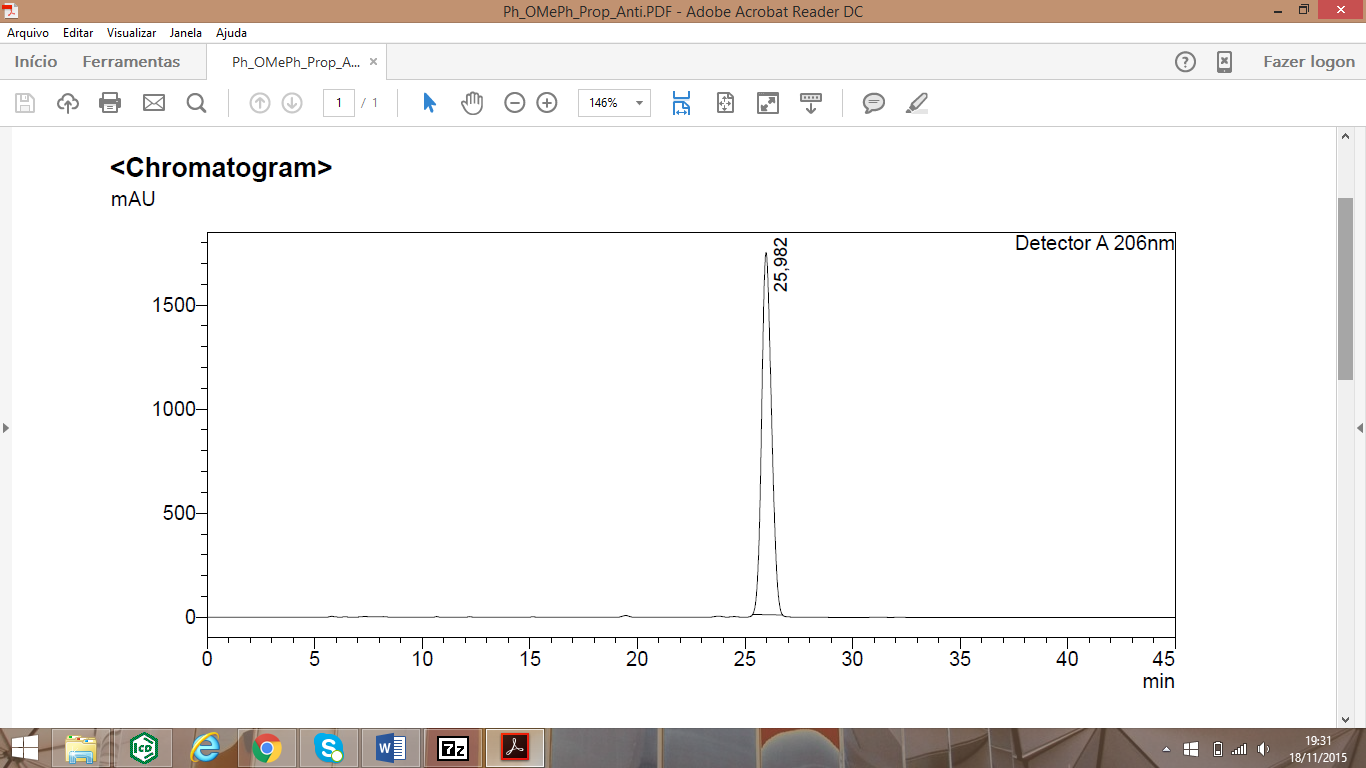


**S8 Fig.** Chromatogram after the HPLC separation to compound **6**

Supplement: S8 Fig — (DOCX) [file pntd.0005343.s008.docx]
