# Supplementary material for: Anti-trypanosomal activity of non-peptidic nitrile-based cysteine protease inhibitors
Source: PLoS Negl Trop Dis. 2017 Feb 21;11(2):e0005343. doi: 10.1371/journal.pntd.0005343 (PMC5344518; doi:10.1371/journal.pntd.0005343)
Supplement: S9 Fig — (DOCX) [file pntd.0005343.s009.docx]

 **S9 Fig.** Concentration response of trypanocidal activity (c = concentration; M = mol/dm^3^)
