# Supplementary material for: Anti-trypanosomal activity of non-peptidic nitrile-based cysteine protease inhibitors
Source: PLoS Negl Trop Dis. 2017 Feb 21;11(2):e0005343. doi: 10.1371/journal.pntd.0005343 (PMC5344518; doi:10.1371/journal.pntd.0005343)
Supplement: S1 Table — (DOCX) [file pntd.0005343.s010.docx]

**S1 Table.** Chromatogram data for S5 Fig.

| Peak | Retention time (min) | Area (%) |
| --- | --- | --- |
| 1 | 17.500 | 80.596 |
| 2 | 21.115 | 19.404 |
| Total |  | 100.000 |
