# Supplementary material for: Anti-trypanosomal activity of non-peptidic nitrile-based cysteine protease inhibitors
Source: PLoS Negl Trop Dis. 2017 Feb 21;11(2):e0005343. doi: 10.1371/journal.pntd.0005343 (PMC5344518; doi:10.1371/journal.pntd.0005343)
Supplement: S2 Table — (DOCX) [file pntd.0005343.s011.docx]

**S2 Table.** Chromatogram data for S6 Fig

| Peak | Retention time (min) | Area (%) |
| --- | --- | --- |
| 1 | 20.370 | 100.00 |
| Total |  | 100.00 |
