# Supplementary material for: Anti-trypanosomal activity of non-peptidic nitrile-based cysteine protease inhibitors
Source: PLoS Negl Trop Dis. 2017 Feb 21;11(2):e0005343. doi: 10.1371/journal.pntd.0005343 (PMC5344518; doi:10.1371/journal.pntd.0005343)
Supplement: S3 Table — (DOCX) [file pntd.0005343.s012.docx]

**S3 Table.** Chromatogram data for S7 Fig.

| Peak | Retention time (min) | Area (%) |
| --- | --- | --- |
| 1 | 18.946 | 6.632 |
| 2 | 25.261 | 93.368 |
