# Supplementary material for: Anti-trypanosomal activity of non-peptidic nitrile-based cysteine protease inhibitors
Source: PLoS Negl Trop Dis. 2017 Feb 21;11(2):e0005343. doi: 10.1371/journal.pntd.0005343 (PMC5344518; doi:10.1371/journal.pntd.0005343)
Supplement: S4 Table — (DOCX) [file pntd.0005343.s013.docx]

**S4 Table.** Chromatogram data for S8 Fig.

| Peak | Retention time (min) | Area (%) |
| --- | --- | --- |
| 1 | 25.982 | 100.00 |
| Total |  | 100.00 |
